# Development and evaluation of two open-source nnU-Net models for automatic segmentation of lung tumors on PET and CT images with and without respiratory motion compensation

**DOI:** 10.1007/s00330-024-10751-2

**Published:** 2024-04-25

**Authors:** Montserrat Carles, Dejan Kuhn, Tobias Fechter, Dimos Baltas, Michael Mix, Ursula Nestle, Anca L. Grosu, Luis Martí-Bonmatí, Gianluca Radicioni, Eleni Gkika

**Affiliations:** 1 La Fe Health Research Institute, Biomedical Imaging Research Group (GIBI230-PREBI) and Imaging La Fe node at Distributed Network for Biomedical Imaging (ReDIB) Unique Scientific and Technical Infra-structures (ICTS), Valencia, Spain; 2 Department of Radiation Oncology, Division of Medical Physics, University Medical Center Freiburg, Faculty of Medicine, Freiburg, Germany; 3 German Cancer Consortium (DKTK), German Cancer Research Center (DKFZ), Partner Site Freiburg, German Cancer Research Center (DKFZ), Heidelberg, Germany; 4 Department of Nuclear Medicine, Faculty of Medicine, University Medical Center Freiburg, Freiburg, Germany; 5 Department of Radiation Oncology, Faculty of Medicine, University Medical Center Freiburg, Freiburg, Germany; 6 Department of Radiation Oncology, Kliniken Maria Hilf GmbH Moenchengladbach, Moechengladbach, Germany

**Keywords:** Lung cancer, Positron emission tomography, Computed tomography, Deep learning, Respiratory motion

## Abstract

**Objectives:**

In lung cancer, one of the main limitations for the optimal integration of the biological and anatomical information derived from Positron Emission Tomography (PET) and Computed Tomography (CT) is the time and expertise required for the evaluation of the different respiratory phases. In this study, we present two open-source models able to automatically segment lung tumors on PET and CT, with and without motion compensation.

**Materials and methods:**

This study involved time-bin gated (4D) and non-gated (3D) PET/CT images from two prospective lung cancer cohorts (Trials 108237 and 108472) and one retrospective. For model construction, the ground truth (GT) was defined by consensus of two experts, and the nnU-Net with 5-fold cross-validation was applied to 560 4D-images for PET and 100 3D-images for CT. The test sets included 270 4D- images and 19 3D-images for PET and 80 4D-images and 27 3D-images for CT, recruited at 10 different centres.

**Results:**

In the performance evaluation with the multicentre test sets, the Dice Similarity Coefficients (DSC) obtained for our PET model were DSC(4D-PET) = 0.74 ± 0.06, improving 19% relative to the DSC between experts and DSC(3D-PET) = 0.82 ± 0.11. The performance for CT was DSC(4D-CT) = 0.61 ± 0.28 and DSC(3D-CT) = 0.63 ± 0.34, improving 4% and 15% relative to DSC between experts.

**Conclusions:**

Performance evaluation demonstrated that the automatic segmentation models have the potential to achieve accuracy comparable to manual segmentation and thus hold promise for clinical application. The resulting models can be freely downloaded and employed to support the integration of 3D- or 4D- PET/CT and to facilitate the evaluation of its impact on lung cancer clinical practice.

**Clinical relevance statement:**

We provide two open-source nnU-Net models for the automatic segmentation of lung tumors on PET/CT to facilitate the optimal integration of biological and anatomical information in clinical practice. The models have superior performance compared to the variability observed in manual segmentations by the different experts for images with and without motion compensation, allowing to take advantage in the clinical practice of the more accurate and robust 4D-quantification.

**Key Points:**

- *Lung tumor segmentation on PET/CT imaging is limited by respiratory motion and manual delineation is time consuming and suffer from inter- and intra-variability*.
- *Our segmentation models had superior performance compared to the manual segmentations by different experts*.
- *Automating PET image segmentation allows for easier clinical implementation of biological information*.

## Introduction

In lung cancer, the ability of Positron Emission Tomography (PET) with [18F]fluoro-2-deoxy-D-glucose (FDG) to exploit the biochemical differences between normal and neoplastic tissue [1] has been proven to be a valuable tool for tumor detection [2], staging [3], treatment planning [4], monitoring [5] and outcome prediction [6, 7]. Based on the sensitivity and specificity implied by FDG-PET imaging in NSCLC [8], the integration of FDG imaging in radiation therapy (RT) clinical practice, which has been conventionally CT-only based, has been recommended [9]. Recent studies have evaluated the role of [^18^F]FDG PET/CT in the precise definition not only of the target volume, but also of subvolumes aiming at a further dose escalation [10–13]. However, the evaluation of the lesion by multimodality PET and Computed Tomography (CT) imaging presents challenges due to respiratory movement [14]. Tumor motion due to the various breathing cycles involved during PET scan acquisition results in inaccurate quantification of tracer distribution, including erroneous estimation of the shape, volume and location of the lesion. Moreover, the different acquisition times of the two studies (few seconds for a CT and several minutes for PET) can result in a spatial mismatch, with artefacts originated by the use of CT images for attenuation correction in PET image reconstruction. Different strategies have been proposed for the management of respiratory motion in PET/CT systems [15–17], such as retrospectively respiratory gated (4D)-PET/CT [18]. As a result of this data processing, an improvement in the image quality and accuracy of estimation of the tracer concentration and distribution should be obtained by compensating for motion effects [19, 20]. The definition of the target volume in RT includes safety margins around the tumor given by geometrical uncertainties (as for example, respiratory motion [21, 22]). The aim of these safety margins is to minimise tumor underdosage. 4D-protocols have been proven to decrease geometrical uncertainties due to respiratory motion and consequently, their corresponding safety margins could be reduced. Therefore, 4D-protocols would lead to a smaller target volume definition and the dose delivered at the organs at risk in the vicinity could be minimised, facilitating dose escalation. In addition, 4D-protocols have been demonstrated to significantly improve the reproducibility of radiomics features [23, 24].

Manual contouring is the method most widely employed for tumor segmentation in RT target delineation, monitoring and radiomics. For these applications, higher accuracy and reproducibility are required beyond what is required in diagnosis. Disadvantages related to the manual approach are to be very laborious and time consuming, especially for the number of images involved in 4D-protocols and to lead to a significant inter- and intra-variability even among experts. An alternative approach consists in relying on automatic or semi-automatic segmentation methods. PET segmentation methods have been proposed [25], ranging from simple uptake thresholding to very elaborate probabilistic models. Applications of artificial intelligence (AI, machine/deep learning) are extremely wide and promising [26, 27]. Superiority of these methods in image segmentation has been shown in many studies [28]. Although the number of automatic AI segmentation algorithms for lung cancer is increasing, most of them focus on CT or MR images [29, 30]. In addition, the AI segmentation models already published for PET have rarely involved 4DPET data [31, 32].

In this study, our primary aim was to develop an AI model for lung tumor segmentation on PET images. In order to facilitate the implementation of the PET segmentation in the clinical workflow, a secondary aim was to develop also an AI model for tumor segmentation on CT images, which is the image modality most commonly employed. In addition, to facilitate the simultaneous implementation of both models, the same open-source convolutional-neuronal-network (nnU-Net) was employed. Finally, in order to maximise the probability that the resulting models have good performance independently of imaging systems and protocols (generalizability), image data was collected from ten different institutions with different involved protocols with (4D) and without (3D) respiratory motion compensation.

## Materials and methods

### Patient cohorts and data sets

Our study included two prospective (PET-Plan and STRIPE) and one retrospective (RC) cohort of lung cancer patients. All patients gave written informed consent according to institutional and federal guidelines. The institutional ethics committee approved the study protocol (EK-Nr 21-1228-S1-retro, EK Nr 108237, EK 113/12, EK-Nr Nr. 108472).

The main clinical characteristics of each trial are described in the following subsections.

#### PET-Plan trial

The randomised controlled PET-Plan trial (ARO 2009-09, NCT00697333, Deutsche Krebshilfe, German Cancer Aid Organisation, Nr 108237) involved patients older than 18 years with histologically or cytologically proven inoperable stage II or III NSCLC. Eligibility criteria also included having an Eastern Cooperative Oncology Group performance status of less than 3; having adequate pulmonary, cardiac, renal and haematological function and being suitable for chemoradiotherapy. The PET-Plan trial was conducted in 24 centres in Germany, Austria and Switzerland, in accordance with the Declaration of Helsinki. Study design, procedures and main outcome results have been published elsewhere [9].

#### STRIPE trial

The prospective monocentre phase II STRIPE trial (Deutsche Krebshilfe, German Cancer Aid Organisation, Nr 108472) involved patients with pulmonary lesions with a maximum diameter of 5 cm (early stage NSCLC or less than 2 pulmonary metastases of a controlled primary tumor), refusing surgery or inoperable due to comorbidities. The study was performed at Medical University Freiburg and the design, procedures and main outcome results have been published elsewhere [33].

#### Retrospective cohort

For the retrospective cohort (RC), patients with primary early-stage (N)SCLC or isolated pulmonary metastases were recruited at the Medical University Freiburg between December 2019 and July 2020. All patients were older than 18 years and showed a maximum of two FDG-PET positive pulmonary lesions with a maximum diameter of 5 cm.

**Fig. 1:**
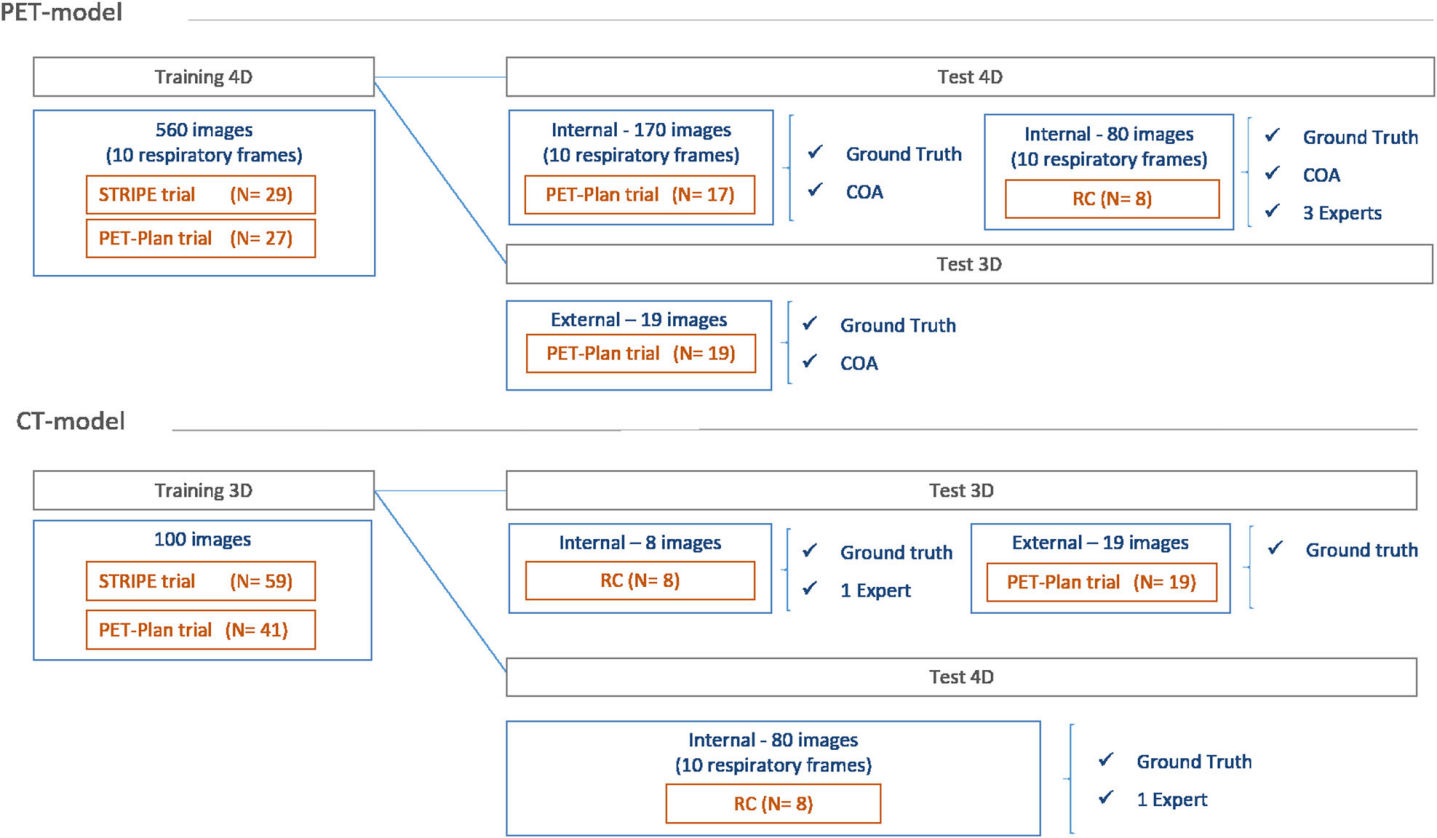
Representation of the training and test sets with (4D) and without (3D) respiratory motion compensated images. External refers to the sets with patients scanned at institutions different from the one in which training patients were scanned

Patients from these three cohorts were split into the training and test cohorts. A representation of the cohorts division employed for the development of CT and PET models is shown in Fig. 1. The training set involved 27 patients from PET-Plan trial and 29 from STRIPE for PET model and 41 patients from PET-Plan trial and 59 from STRIPE for CT model, all of them recruited in Medical Centre Freiburg. The performance of the models was independently tested on a multicentre cohort, which includes prospective and retrospective patients: 36 patients from PET-Plan and 8 from retrospective cohort for PET model and 19 patients from PET-Plan trial and 8 from retrospective cohort for CT model.

### PET/CT acquisition

After a fasting period of at least 4 h (glucose level less than 150 mg/dl), all patients underwent a diagnostic whole-body [^18^F]FDG PET/CT scan at approximately 60 min following weight-adjusted [^18^F]FDG intravenous injection.

All patients imaged at University Medical Centre Freiburg underwent a chest-limited 4D PET/CT scan for one bed position (15 min acquisition time) at approximately 102 min after FDG injection. Patient respiration was monitored with a belt (Mayo Clinic Respiratory feedback system) and the respiratory curve was synchronised with the acquisition time of scanner. The data was reconstructed in 10 time-based [34] respiratory phases with a fully 3D list mode LOR TOF algorithm, involving a relaxed List Mode Ordered Subset (BLOB-OS-TF), resulting in 10 PET and 10 CT sets. 4DPET was reconstructed in a 144 × 144 matrix and 4 × 4 × 4 mm³ voxel size and the 4DCT with 512 × 512 matrix and 1.17 × 1.17 × 2 mm³ voxel size. Attenuation correction of 4DPET was based on the corresponding respiratory-phase 4DCT. Correction for scatter and randoms was applied and the standard uptake value (SUV) was calculated with body weight.

At the University Medical Centre Freiburg scans were performed on two different PET/CT systems from Philips: GEMINI TF TOF 64 (TF64) and GEMINI TF 16 Big Bore (BB). The imaging systems employed in the other nine institutions were: two GEMINI TF TOF 16 from Philips, two SIEMENS Biograph 64, two SIEMENS Biograph 40 mCT, one SIEMENS Biograph 128, one Alegro Body from Philips and one Guardian Body from Philips. The scanners fulfilled the requirements indicated in the European Association of Nuclear Medicine (EANM) imaging guidelines and obtained EANM Research Ltd. (EARL) accreditation. PET image voxel size was 4 × 4 × 4 mm^3^ for eight patients, 4 × 4 × 5 mm^3^ for seven patients and 2.65 × 2.65 × 3.37 mm^3^ for three patients. In CT, voxel sizes varied more significantly across institutions, with a thickness ranging from 1 to 5 mm and transaxial pixel ranging from 0.63 × 0.63 mm^2^ to 1.37 × 1.37 mm^2^.

### Segmentation approaches employed for training and validation

#### Tumor manual segmentation

Manual segmentation of the primary tumor and lymph nodes based on PET and CT was performed according to the clinical study protocol and current guidelines for contouring with standardised window-level [35]: on CT the pre-set window should range from 600 to 1600 HU for tumours surrounded by lung tissue and from 20 to 400 for lymph nodes and primary tumours invading the mediastinum or chest wall. For PET a window with standardised-uptake-values ranging from 2 to 10 was recommended.

Ground truth was defined by the consensus of two radiation oncologists. One additional expert contoured the 3D- and 4D-CT sets for the eight patients for the internal validation of CT. Three additional experts contoured each of the time-bins for the eight patients for the retrospective cohort employed in the internal evaluation of 4D-PET algorithm. Besides technical instructions for the use of the delineation software, the readers received no further assistance and were blinded to all other delineations.

#### PET semiautomatic contrast-oriented algorithm

The semiautomatic approach employed for PET segmentation was based on the application of an adaptive threshold taking into account the contrast between tumor concentration (mean value for a 70% isocontour of maximum intensity within the lesion) and background (automatically derived from the whole image) [36]. The semi-automatic character of this approach relied on the fact that the user was required to indicate a tumor voxel (seed) and maximum intensity was automatically identified in its neighborhood. In order to avoid disconnected regions, a threshold was applied with a six-neighborhood 3D region-growing algorithm from the seed. This approach required phantom-based calibration.

### nn-UNet

The nnU-Net [37] was employed for the development of (i) a PET segmentation model, trained with 560 PET respiratory motion compensated images and their corresponding ground truth segmentations and (ii) a CT segmentation model, trained with 100 CT non-motion compensated images and their corresponding ground truth segmentations. nnU-Net is a deep learning-based segmentation method that automatically configures itself, including preprocessing, network architecture, training and post-processing. nnU-Net has a U-Net architecture with plain convolutions, z-score normalisation, leaky ReLU and two blocks (encoder-decoder) per resolution stage. From the three configurations available, the 3D U-Net with full image resolution was employed in this study. Based on the results from a 5-fold cross-validation, the best model was ensemble. The nnU-Net [37] and the resulting models (GitHub_PETCTLungSegmentationModels_Link) can be free download.

### Metrics for accuracy evaluation

We evaluated the agreement between segmented volumes by computing Dice Similarity Coefficient (DSC) [38], which represents the size of the union of two volumes and the formula is 2(A ∩ B)/(A + B), where A is the ground truth and B the segmented volume. We additionally computed the Positive Predictive Value (PPV) in order to account for the cases in which one set of segmented volumes tends to be included within the other set [36]. The formula for PPV is (A ∩ B)/B = True-Positive/(True-Positive + False-Positive). The Hausdorff distance [39], which represents the maximum distance between contours surfaces is of interest in order to identify distant regions incorrectly classified as tumour.

## Results

### PET segmentation model

**Fig. 2:**
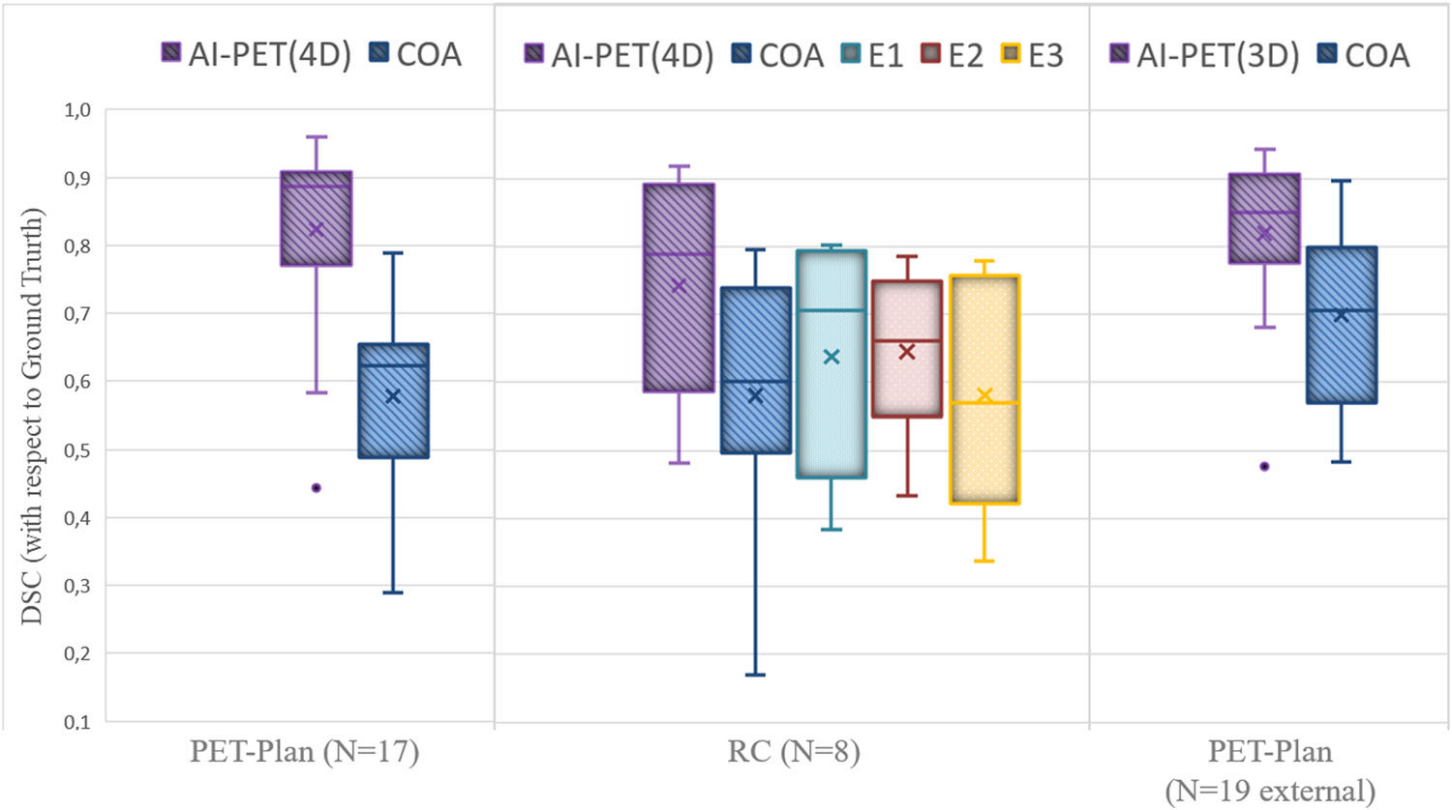
DSC for the different PET segmentations approaches (artificial- intelligence-algorithm AI, contrast-oriented-algorithm COA and experts E) with respect to the ground truth defined by consensus of two radiation oncologists. Performance for the segmentation model on PET images with (4D) and without (3D) respiratory motion compensation are presented. The average value is represented by the cross and the median by the line

After a 5-fold cross-validation training with 560 4D-PET images, the resulting optimal PET segmentation model was tested on three test sets, being the ground truth defined by the same radiation oncologists as in the training data. The accuracy of the segmentation provided by the nnU-Net PET model, with respect to the GT, was compared with the accuracy for the semiautomatic contrast-oriented-algorithm (COA) and the accuracy for the segmentation performed by other experts. Results for DSC are shown in Fig. 2.

**Fig. 3:**
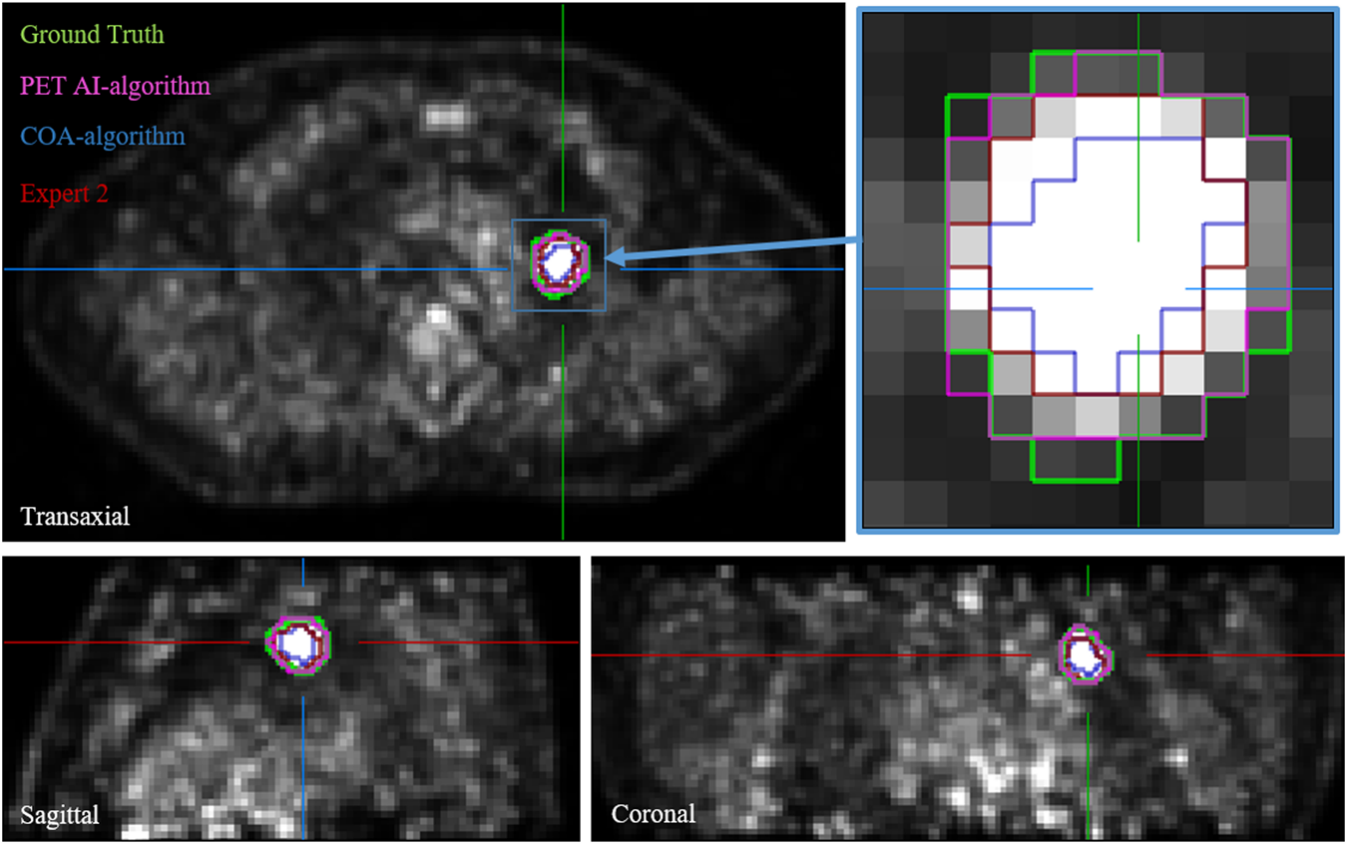
Different PET segmentations approaches (ground truth, artificial intelligence-algorithm AI, contrast-oriented-algorithm COA and expert with the best DSC with respect to the ground truth) for a patient employed in the validation of the performance of the algorithm on PET images with respiratory motion compensation

First, the model was tested with the 170 4D-PET images of patients involved in the prospective PET-Plan trial, the DSC(4D-PET) was 0.83 ± 0.13, improving 43% with respect to the accuracy for the COA-algorithm, DSC(4D-COA) = 0.58 ± 0.15. Second, the model was tested with the 80 4D-PET images of patients involved in the retrospective cohort, the DSC(4D-PET) was 0.74 ± 0.06, improving 28% with respect to the accuracy for the COA-algorithm with DSC(4D-COA) = 0.58 ± 0.08 and improving 16%, 16% and 28% with respect to the accuracy for the three other experts, with DSC(4D-Expert1) = 0.64 ± 0.06, DSC(4D-Expert2) = 0.64 ± 0.09 and DSC(4D-Expert3) = 0.58 ± 0.06. In Fig. 2 it is shown that the model accuracy for the patient of this cohort with the best performance, DSC(4D-PET) = 0.92, was better than for COA (0.79) and for the three experts (0.80, 0.78 and 0.78) and the DSC for the patient with worst performance by the AI-model (0.48) was also better than for COA (0.38) and experts (0.38, 0.43 and 0.34). In addition, in order to reject distant false positives, the Housdorff distance was computed. Values were comparable across the different approaches 2.1 ± 0.7 for AI-model, 2.5 ± 0.5 for COA and 2.4 ± 0.4, 2.4 ± 0.5 and 2.6 ± 0.4 for the three experts. An example of the different PET tumor segmentation approaches is shown in Fig. 3.

**Fig. 4:**
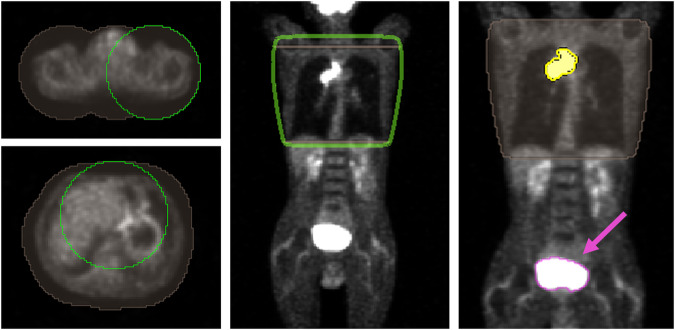
Workflow for the lung mask creation: **left** regions of interest (brown) are manually delineated surrounding the body by a 50 voxel circle (green) in two slices located up and down the lung region; **middle** automatic interpolation is performed between the slices and lung region mask resulted (green) and (**right**) boolean intersection (yellow) between the AI segmentation and the lung region mask is obtained and consequently, the bladder region is rejected (segmentation pointed by the pink arrow)

Finally, the model was tested with the 19 3D-PET images of patients prospectively recruited in 9 different centres involved in the PET-Plan trial. DSC(3D-PET) was 0.83 ± 0.11, improving 19% with respect to the accuracy for the COA-algorithm, with DSC(4D-COA) = 0.70 ± 0.12. It should be remarked that for patients who received whole-body PET scans, the AI-model also segmented the bladder and other tracer-enhanced pelvic structures. This limitation was avoided by a simple lung region restriction manually performed by interpolation of two regions of interest, Fig. 4. The time invested for this lung mask was comparable to the time required to place the seeds for the COA algorithm (< 1 min).

### CT segmentation model

**Fig. 5:**
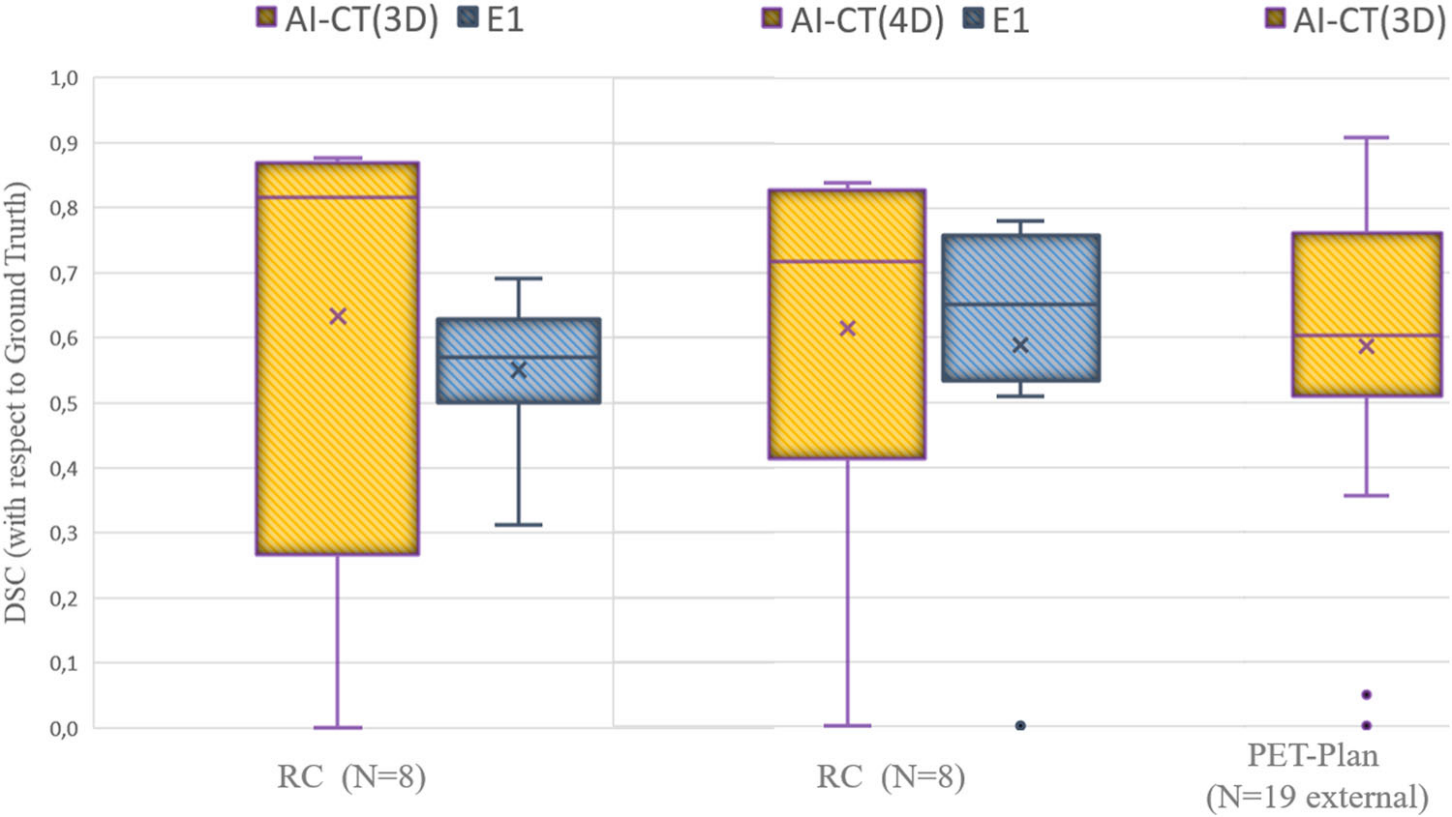
DSC of the different CT segmentation approaches (artificial- intelligence-algorithm AI and expert) with respect to the ground truth defined by consensus of two radiation oncologists. Performance for the segmentation model on CT images with (4D) and without (3D) respiratory motion compensation are presented. The average value is represented by the cross and the median by the line

After a 5-fold cross-validation training with 100 3D-CT images, the resulted optimal CT segmentation model was tested on three test sets. In Fig. 5, values for the DSC are shown.

**Fig. 6:**
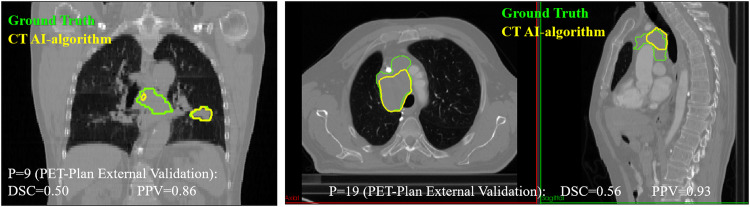
Different CT segmentation approaches for two PET-Plan patients involved in the external validation

First, the model was tested with the 8 3D-CT images of patients retrospectively recruited. DSC(3D-CT) was 0.63 ± 0.34, improving 15% with respect to the experts DSC(3D-Expert) = 0.55 ± 0.11. The nnU-Net model showed an accuracy higher than 0.69, for all patients apart from two. The two DSC values for these two patients (0.12 and 0) are responsible of the difference between the average value (0.63 box cross in Fig. 5) and the median value (0.81, box line in Fig. 5). Second, the model was tested with the 19 3D-CT images of patients prospectively recruited in 9 different centres involved in the PET-Plan trial. DSC(3D-CT) was 0.59 ± 0.24. Overall, a trend of good detection but an underestimation of the size of the lesion was observed. This trend was confirmed by an average value of positive-predictive-value of 0.77 ± 0.23. In Fig. 6 examples of contours for two patients are shown, being DSC and PPV values also provided.

Finally, the model was tested with the 80 4D-CT images of patients involved in the prospective PET-Plan cohort, the DSC(4D-CT) was 0.61 ± 0.28, improving 4% with respect to the accuracy for the expert, with DSC(3D-Expert) = 0.59 ± 0.24. DSC = 0 for the expert data corresponding to a patient for which one of the experts considered image quality too poor in order to delineate the lesion. For this patient, an algorithm was able to detect the lesion, but segmentation accuracy was very poor (DSC = 0.33).

## Discussion

In this study, we present a completely automatic algorithm to segment lung cancer tumors on PET images, with and without respiratory motion compensation. The performance of the algorithm has been evaluated with patients of ten different centres and by comparison with respect to a previously evaluated semiautomatic algorithm and with respect to the variability observed across different experts. In order to facilitate its clinical implementation, an automatic method for tumor segmentation on CT images, with and without motion compensation, based on the same nnU-Net has been additionally developed. The convolutional–network employed by training [38] and the resulting models (GitHub_PETCTLungSegmentationModels_Link) are open source.

One of the clinical applications for the resulting models would be their use for RT planning and monitoring in lung cancer. According to the ESTRO-ACROP guidelines for target volume (TV) definition in the treatment of locally advanced non-small cell lung cancer, both a contrast-enhanced diagnostic CT scan and whole-body diagnostic FDG-PET-CT are considered mandatory in preparation for TV delineation for curative RT or chemo-RT [38]. Typically, the definition of the target volume is performed on the CT, adding the information derived from PET. Assessment of respiratory motion on a respiratory-correlated 4D CT scan is also recommended. 4D-gated PET/CT in the treatment position is optional but may improve the sensitivity of nodal identification and provide additional valuable information to help differentiate between tumor extent and adjacent tissues [39]. By applying our resulting models, the information from both images could be automatically integrated. Furthermore, with the advantage of the enhanced accuracy and robustness provided by 4D-protocolos, these segmentations could be employed to improve the TV definition, optimising therefore treatment planning and monitoring.

Most of the previous convolutional-neural-network (CNN) approaches for lung tumor segmentation have been based on CT images [40–42] or on MR images [43] with 330, 1210, 19 and 9 patients, respectively. The benefit of PET imaging for lung cancer patients has been already demonstrated and consequently, segmentation models based on PET/CT images have been developed [44, 45], with 84 and 32 patients, respectively. It is known that the performance of AI segmentation models depends on the availability of a large amount of data for its training step. In PET, it is usually challenging to gather such large cohorts of patients compared to other imaging modalities [29]. In our study, the size of the PET cohort (100 patients) is larger than the size of the cohorts commonly employed for PET segmentation model development and validation [46]. It is also known that segmentation CNN-based approaches are used to perform well during training but demonstrate reduced performance during the validation and test steps. Therefore, cross-validation and testing of the model performance have been strongly recommended [29]. Our initial training cohort of 56 patients has been 5-fold cross-validated in order to take more benefit from the limited size of the sample. The performance of the resulting model after cross-validation, has been tested with 25 patients prospectively recruited at the same centre. Because the level of performance of AI models, as well as their reproducibility and robustness, is sensitive to imaging protocols and devices, we additionally evaluated the performance of our model for 19 patients prospectively recruited at 9 other centres. Segmentation accuracy in terms of DSC values was comparable for both, internal and external cohorts, allowing concluding an acceptable generalizability of the model. In terms of the clinical acceptability, it is important to include a comparison of the algorithm performance with respect to the manual contours and the variability across experts, which is the standard method commonly employed in our clinical practice for tumor segmentation. In contrast to most of the previous publications, our study not only includes the comparison with respect to the established ground truth (consensus of two experts), but the analysis also involved the manual contours of three additional experts. From the results in this comparison, it could be concluded that the accuracy of the proposed model is promising in terms of robustness. In terms of the viability of its clinical implementation, a CT-based segmentation model has been developed with the same nnU-Net in order to facilitate the integration of PET segmentation model in the clinical workflow. Although the average values of DSC for the resulting CT model were not high, the performance of the algorithm was proved to be better than the variability observed among experts. Finally, previous literature has demonstrated that the compensation of respiratory motion improves accuracy and reproducibility for the biomarker distribution quantification [20, 23, 24]. However, 4D-PET imaging implementation in the clinical workflow is hindered by the time and expertise, required from the clinical site, for a proper analysis and integration of the functional information. The segmentation algorithm resulted from our study has been proven to accurately segment tumors with both imaging protocols, with (4D) and without (3D) motion compensation. To evaluate the impact of motion compensation on the performance of our model, images with and without motion compensation for the same patients should be compared. Unfortunately, only for 8 patients and only for CT images, both imaging protocols (3D and 4D) were available. For these patients, the better performance obtained for the model with non-compensated images (DSC(3D-CT) = 0.63 ± 0.34, improving 15% improvement relative to experts) than with motion-compensated images (DSC(4D-CT) = 0.61 ± 0.28, improving 4% relative to experts), could be justified by the fact that the model has been trained with non-compensated images. It is not the case for the manual contours, where the accuracy with motion-compensated images, DSC(4D-Expert) = 0.59 ± 0.24, was better than without motion compensation, DSC(3D-Expert) = 0.55 ± 0.11. However, the accuracy for the nnU-Net model is better than the variability observed across experts in both 3D and 4D images, supporting the applicability of the model independently of the imaging protocol. Therefore, this segmentation model could facilitate the clinical implementation of 4D-PET images for monitoring and for the development of prognosis models, where the quantification of PET images plays a crucial role in order to identify clinically relevant changes and where, consequently, the accuracy and reproducibility provided by these 4D-protocols are of special interest.

In order to quantify the performance of our models, 3D computation of DSC was employed, because it is the most common and standardised method for the comparison of segmentations in medical imaging [38]. However, a high DSC does not necessarily indicate good agreement. An example could be a prediction of a tumor similar in size and location to the ground truth, but with a distant small region incorrectly classified also as a tumor. This scenario is likely to be found in lung tumor segmentation on 4D-PET thorax images, because of the high uptake of the heart and the noise implied by respiratory frames. For these reasons, Hausdorff distance [39] was additionally computed, and the results allowed us to confirm the good performance of our model. In addition, a positive-predictive-value [36] permitted to quantify the qualitatively observed trend in CT model performance, i.e., a high positive-predictive-value demonstrated that the model is able to detect the lesion and its location, but underestimates its size.

The main advantages of nnU-Net are that a preprocessing of the input data is not required, the non-fixed parameters for the training process of the model are automatically configured based on the input image data and the resulting model is generated and optimised without user interaction. In addition, the good performance of the nnU-Net method has been proved in different data sets and the code can be free to download [37]. For these reasons, the whole process can be easily reproduced. Medical data sharing is one of the most important concerns in AI development. It facilitates the comparison of the performance for different algorithms, can be used for the improvement of the generalizability of the algorithms, and makes feasible for developers to test their new CNNs. The main limitation in our study was that the clinical data employed is not open access. There are different CT and MR image repositories for lung cancer. The Lung Image Database Consortium-Image Database Resource Initiative (LIDC) is the world’s largest publicly available database and contains 1018 CT scans of 1010 patients. The Automatic Nodule Detection 2009 (ANODE09) is another publicly available database consisting of 55 CT scans, annotated by two radiologists. Also, the TIME [47]; ELCAP [48] and LISS [49], are available in the public domain for lung nodule research. Future work should focus on PET image repositories that will also facilitate the development of PET AI tools for its use in clinical practice.

## Conclusion

We presented two open-source nnU-Net models for lung tumor segmentation on PET and CT images, with and without respiratory motion compensation. Performance evaluation demonstrated that the automatic segmentation models have the potential to achieve accuracy comparable to manual segmentation and thus hold promise for clinical application. They could therefore be employed to facilitate the integration of FDG-PET/CT in lung cancer and to take advantage of the better quality provided by 4D quantification.

## Data Availability

The resulted segmentation models can be downloaded (GitHub_PETCTLungSegmentationModels_Link).

## List of abbreviations

3D: Without motion compensation
4D: With motion compensation - respiratory gated
AI: Artificial Intelligence
CNN: Convolutional-neural-network
COA: Contrast-oriented-algorithm
CT: Computed tomography
EANM: European Association of Nuclear Medicine
FDG: [18F]fluoro-2-deoxy-D-glucose
(N)SCLC: Non-small cell lung cancer
PET: Positron emission tomography
RC: Retrospective cohort
RT: Radiation therapy
TV: Target volume
