# Correction: Development and evaluation of two open-source nnU-Net models for automatic segmentation of lung tumors on PET and CT images with and without respiratory motion compensation

**DOI:** 10.1007/s00330-024-10826-0

**Published:** 2024-06-18

**Authors:** Montserrat Carles, Dejan Kuhn, Tobias Fechter, Dimos Baltas, Michael Mix, Ursula Nestle, Anca L. Grosu, Luis Martí-Bonmatí, Gianluca Radicioni, Eleni Gkika

**Affiliations:** 1 La Fe Health Research Institute, Biomedical Imaging Research Group (GIBI230-PREBI) and Imaging La Fe node at Distributed Network for Biomedical Imaging (ReDIB) Unique Scientific and Technical Infra-structures (ICTS), Valencia, Spain; 2 Division of Medical Physics, Department of Radiation Oncology, Faculty of Medicine, University Medical Center Freiburg, Freiburg, Germany; 3 German Cancer Consortium (DKTK), German Cancer Research Center (DKFZ), Partner Site Freiburg, German Cancer Research Center (DKFZ), Heidelberg, Germany; 4 Department of Nuclear Medicine, Faculty of Medicine, University Medical Center Freiburg, Freiburg, Germany; 5 Department of Radiation Oncology, Faculty of Medicine, University Medical Center Freiburg, Freiburg, Germany; 6 Department of Radiation Oncology, Kliniken Maria Hilf GmbH Moenchengladbach, Moechengladbach, Germany

**Correction to: European Radiology**

10.1007/s00330-024-10751-2; published online 25 April 2024

In the original version of this article, the following link has not been functioning as intended:

GitHub_PETCTLungSegmentationModels_Link/ (https://github.com/MonCarFa/PET-CT-Lung-Segmentation-Models/)

The original publication has been corrected.

